# Randomized Controlled Field Trial to Assess the Immunogenicity and Safety of Rift Valley Fever Clone 13 Vaccine in Livestock

**DOI:** 10.1371/journal.pntd.0003550

**Published:** 2015-03-10

**Authors:** M. Kariuki Njenga, Leonard Njagi, S. Mwangi Thumbi, Samuel Kahariri, Jane Githinji, Eunice Omondi, Amy Baden, Mbabu Murithi, Janusz Paweska, Peter M. Ithondeka, Kisa J. Ngeiywa, Baptiste Dungu, Meritxell Donadeu, Peninah M. Munyua

**Affiliations:** 1 Division of Global Health Protection, United States Centers for Disease Control and Prevention-Kenya, Nairobi, Kenya; 2 Paul G. Allen School for Global Animal Health, Washington State University, Pullman, Washington, United States of America; 3 Kenya Ministry of Agriculture Livestock and Fisheries, Nairobi, Kenya; 4 National Institute for Communicable Diseases, Pretoria, South Africa; 5 Global Alliance for Livestock Veterinary Medicines, Edinburg, Scotland, United Kingdom; University of Pittsburgh, UNITED STATES

## Abstract

**Background:**

Although livestock vaccination is effective in preventing Rift Valley fever (RVF) epidemics, there are concerns about safety and effectiveness of the only commercially available RVF Smithburn vaccine. We conducted a randomized controlled field trial to evaluate the immunogenicity and safety of the new RVF Clone 13 vaccine, recently registered in South Africa.

**Methods:**

In a blinded randomized controlled field trial, 404 animals (85 cattle, 168 sheep, and 151 goats) in three farms in Kenya were divided into three groups. Group A included males and non-pregnant females that were randomized and assigned to two groups; one vaccinated with RVF Clone 13 and the other given placebo. Groups B included animals in 1^st^ half of pregnancy, and group C animals in 2^nd^ half of pregnancy, which were also randomized and either vaccinated and given placebo. Animals were monitored for one year and virus antibodies titers assessed on days 14, 28, 56, 183 and 365.

**Results:**

In vaccinated goats (N = 72), 72% developed anti-RVF virus IgM antibodies and 97% neutralizing IgG antibodies. In vaccinated sheep (N = 77), 84% developed IgM and 91% neutralizing IgG antibodies. Vaccinated cattle (N = 42) did not develop IgM antibodies but 67% developed neutralizing IgG antibodies. At day 14 post-vaccination, the odds of being seropositive for IgG in the vaccine group was 3.6 (95% CI, 1.5 – 9.2) in cattle, 90.0 (95% CI, 25.1 – 579.2) in goats, and 40.0 (95% CI, 16.5 – 110.5) in sheep. Abortion was observed in one vaccinated goat but histopathologic analysis did not indicate RVF virus infection. There was no evidence of teratogenicity in vaccinated or placebo animals.

**Conclusions:**

The results suggest RVF Clone 13 vaccine is safe to use and has high (>90%) immunogenicity in sheep and goats but moderate (> 65%) immunogenicity in cattle.

## Author Summary

Although livestock vaccination is effective in preventing Rift Valley fever (RVF) outbreaks, there are concerns about safety and effectiveness of the only commercially available vaccine for the disease. Here, we conducted a field trial in Kenya to evaluate the safety and ability to induce protection for a new RVF vaccine, referred to as Clone 13, that was recently registered in South Africa. A total of 404 animals, consisting of cattle, sheep, and goats, were divided two groups and one group was vaccinated with Clone 13 vaccine while the other group was not vaccinated. The animals were followed for one year and analyzed for RVF antibody levels at days 14, 28, 56, 183, and 365 after vaccination. Between 91% and 97% of vaccinated sheep and goats develop antibodies to the vaccine, whereas only 67% of the vaccinated cattle developed antibodies. These finding indicate that the Clone 13 vaccine induces high levels of protective antibodies in sheep and goats and moderate levels in cattle. The vaccine was safe since none of vaccinated animals developed evidence of RVF disease including deformities in newborns, and only 1 out of 120 pregnant animals had an abortion that was not associated with the RVF disease.

## Introduction

Rift Valley fever (RVF) is an acute disease that is caused by a phlebovirus of the Bunyaviridae family of viruses that affects livestock (cattle, sheep, goats, camels) and humans in Africa and the Arabian Peninsula [1–3]. In Africa, periodic and severe epidemics have been reported in Kenya, Somalia, Tanzania, Sudan, South Africa, Zimbabwe, Senegal, Mauritania, Egypt, and Madagascar [2,4–8]. Even though determining the actual morbidity and mortality in humans has been difficult, an RVF epidemic in Egypt in 1977 resulted in an estimated 200,000 human cases and 600 deaths whereas the one in East Africa (Kenya, Somalia, Tanzania) in 1997–98 resulted in over 100,000 cases and over 450 deaths in Kenya alone [4,9–11]. The RVF epidemic in Saudi Arabia and Yemen in 2002 resulted in an estimated 4000 human cases and over 200 deaths [2,3].

Over 80% of the RVF-infected humans are either asymptomatic or have a mild to moderate influenza-like disease. However most fatal cases develop severe disease characterized by central nervous system complications, retinitis, severe jaundice, haemorrhagic syndrome and death [12,13]. The RVF epidemics also result in massive livestock abortions, death of primarily young animals, and devastating economic losses associated with animal quarantines and trade restrictions [14]. For example, the economic losses resulting from the 2006–07 RVF epidemic in Kenya alone were estimated at US$32 million [15]. The losses were associated with effects on the livestock value chains such as livestock producers, traders, slaughterhouses, and butchers; and effects on the national income such as decline in the livestock market and other sectors such as transportation, chemicals, petroleum and tourism [15].

The RVF epidemics occur during years of El Niño weather characterized by heavier than usual rainfall, resulting in hatching of a high population of floodwater *Aedes* species mosquitoes that transmit the virus to susceptible livestock and human populations [1]. Apart from mosquito transmission, humans can be infected by consuming animal products from infected animals, a transmission pathway that is associated with most of the severe human cases [16]. For the endemic countries, there is a prediction model that has been improving steadily, which can provide an early warning of a few months prior to an epidemic [17,18]. Since the RVF outbreak in the Arabian Peninsula in 2000, there has been increasing concern that RVF virus can be spread to other non-endemic regions through movement of infected travellers, mosquitoes, or animals, which would raise major biosafety and biosecurity concerns globally [19]. Experts agree that the most effective intervention against epidemics and for enhancing preparedness in the face of possible global spread of RVF virus is mass vaccination of livestock in high risk areas, and perhaps humans that may be at immediate risk [20].

If carried out effectively before an epidemic, livestock vaccination can either prevent the epidemic or significantly reduce its extent and severity in livestock and subsequent human infections [21]. In sub-Saharan Africa, vaccination of susceptible livestock is widely used, but with varying degrees of success. Two vaccines are commercially available for vaccination of animals against RVF; both of them produced from a pantropic Entebbe strain of RVF virus referred to as Smithburn strain, that was isolated from mosquitoes in Uganda in 1944 and attenuated through extensive passaging in mice [22–24]. A formalin inactivated Smithburn vaccine is safe for use during epizootics and in pregnant animals; but it is poorly immunogenic and confers only short term immunity, thus requiring booster doses and regular revaccination to achieve and maintain immunity [22]. The inactivated Smithburn vaccine also confers poor colostral immunity and it requires long production lead time due to the tedious process of inactivation that requires rigorous biosafety and biosecurity measures. The live Smithburn vaccine is more immunogenic and requires only a single dose; however it causes abortion and foetal teratogenicity when administered to pregnant animals [23]. In addition, there have been reports that the live vaccine can revert to virulence and be transmitted from vaccinated to susceptible animal and humans resulting in RVF disease [14]. Because of these shortcomings of the Smithburn vaccines, there has been need to develop safer and more efficacious RVF vaccines. Presently, there is no commercially available human vaccine against RVF disease.

There are a number of candidate livestock vaccines that have undergone experimental and field trials. The MP-12 is an RVF virus strain was generated from the wild type parental strain ZH548 (isolated from an infected patient in Egypt) following serial passages with a chemical mutagen. MP-12 is a temperature-sensitive mutant that carries mutations in the M- and L-segments that contribute to the attenuation of virulence [25,26]. There are two candidate MP-12 vaccines, one with a deletion at the NSs region of the virus genome, and the other with a deletion at the NSm region of the virus [27]. In experimental infections in livestock and non-human primates, the MP-12 NSm mutant has shown good immunogenicity and protection from challenge [27,28]. Recently, a recombinant RVF virus derived from RVF strain ZH501, an isolate from a human patient in Egypt, but lacking the NSm gene in the M-segment and carrying a green fluorescent protein in place of the NSs gene in the S-segment (ΔNSs-ΔNSmRVFV) was generated by reverse genetics [29]. The availability of such a vaccine virus lacking virulence-associated genes and insertion of the non-viral GFP gives it the capability for distinguishing vaccinated animals from natural infections, which is an important feature. Experimental studies using ΔNSs-ΔNSmRVFV in sheep showed no virulence even in high doses, accompanied by strong immunogenicity and no teratogeny [29].

The RVF Clone 13 virus is a plaque isolate of the 74HB59 strain of RVF virus recovered from an RVF-infected patient in Central Africa that lacks approximately 70% of a NSs open reading frame, and is therefore significantly attenuated [30]. A live RVF Clone 13 vaccine was registered for use in cattle, sheep and goats in South Africa in 2009 and in Namibia in 2011, and over 50 million doses have been sold since [23]. Studies on safety and immunogenicity of the vaccine carried out in South Africa indicates that the vaccine is highly immunogenic, and it does not cause abortion or foetal teratogenicity in ewes vaccinated during pregnancy [31]. The vaccine was also found to induce production of neutralizing antibodies that were protective against virus challenge in ewes and calves [31,32]. In experimental challenge studies, RVF Clone 13 vaccine protected cattle and sheep to titers similar to those observed for the Smithburn strain [31,32] with a good dose-response effect. More importantly, no abortion or teratogeny were reported in animals vaccinated with RVF Clone 13, in contrast to animals vaccinated with the Smithburn strain vaccine [32].

Here, we carried out field trials for RVF Clone 13 RVF vaccine in commercial livestock farms in Kenya in order to provide more data that would enable this vaccine to gain broader registration in Africa as an important tool in the prevention and control of RVF. Cattle, sheep and goats in three livestock farms, including those in early and late pregnancy, were vaccinated with RVF Clone 13 to determine the immunogenicity and safety of the vaccine at the recommended dose in adult and young animals and in pregnant females at different stages of pregnancy.

## Materials and Methods

### Vaccine

Clone 13 RVF (strain 74HB59) vaccine was provided by Onderstepoort Biological Products (OBP, Onderstepoort, South Africa) as freeze dried pellet (Batch # 13). For injection, the pellet was suspension in 1 milliliter of diluent consisting of glucosamine serum (Batch # 8306). Each reconstituted milliliter contained at least 1 x 10^5^ plaque forming units per ml of the RVF Clone virus, the recommended minimum dose [31]. The placebo was the glucosamine serum diluent in 1 milliliter volume provided by OBP. Each study animals was subcutaneously injected with 1 ml of either reconstituted RVF Clone 13 vaccine or placebo. Cold chain was maintained throughout the transportation of the vaccine from the manufacturer to injection of animals.

### Study Sites and Animals

Ethical approval for this study was obtained from the Kenya Medical Research Institute, Nairobi, Kenya (SSC # 2098) and it adhered to the Kenya national guidelines for animal care and use as stipulated by the National Commission for Science, Technology, and Innovation. Approval was also obtained from the United States’ Centers for Disease Control and Prevention and the Department of Veterinary Services, Government of Kenya. The study was conducted at three livestock farms in Kenya; the Kiboko Farm, Kabete Veterinary Farm, and Ngong Veterinary Farm. The Kiboko farm, owned by the Kenya Agriculture Research Institute is located in Makindu division of Makueni County. The farm size is 15,400 hectares mainly covered by pastures, research crops, fodder and pastures, shrubs and woodland. Wild animals found in the farm include elephants, antelopes, and leopards. The domestic animals kept in the farm are cattle (Sahiwal, Borana and Zebu breeds) and gala goats. The farming system is outdoor grazing for the cattle and goats, which are kept separate. Routine vaccination is carried out against Foot-and-mouth disease for cattle and contagious caprine pleuropneumonia for goats. Weekly anti-tick dipping is carried out for cattle. The Kabete farm, owned by the government of Kenya, is located in Westlands District of Nairobi County. The farm size is about five acres and the animal species kept are cattle (Friesian, Ayrshire and Friesian-Ayrshire crosses), sheep (Dopper crosses) and horses. The farm practices outdoor grazing with hay supplementation. The Ngong farm, owned by the government of Kenya is located in Kajiado North District of Kajiado County. The farm is 1,138 acres and has cattle (Friesian, Ayrshire, Charolaise and their crosses) and sheep (Doper, Red Maasai and their crosses). The farm practices extensive outdoor grazing and routine vaccination is carried out against Foot-and-mouth disease for cattle and Enterotoxaemia and Pestes de Petit Ruminant for sheep. The farms were found suitable due to their good record keeping of breeding, health and feeding records for individual animals. The study was conducted in cattle, sheep and goats aged at least 6 months at the start of the study. Cattle and goats were enrolled in Kiboko farm whereas cattle and sheep were enrolled in both Ngong and Kabete farms. Prior to enrolment into the study, RVF virus sero-status of the animals was determined; only seronegative animals were enrolled. All animals were given individual identification using ear tags.

### Study Design

**Fig 1:**
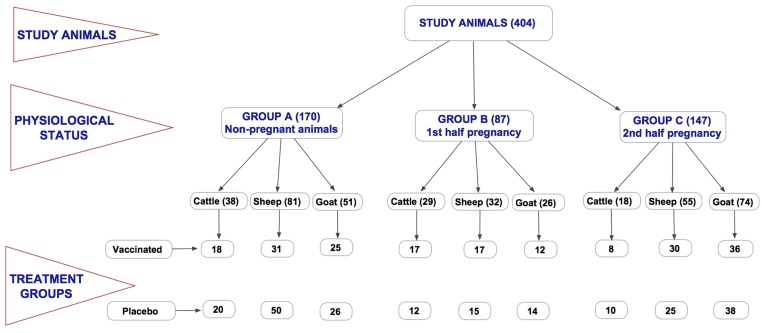
Schematic summary of the study design showing the species, the numbers and the physiological status of the study animals. The study was carried out in three sites (Kabete, Kiboko and Ngong)—all government farms with similar farm management conditions. We used 404 animals in the study, including 85 cattle, 168 sheep, and 151 goats. Of these, 194 were vaccinated with RVF Clone 13 vaccine whereas 210 were injected with placebo. The study animals were divided into 3 groups; Group A included non-pregnant animals, Group B included animal in 1^st^ half of the pregnancy, and Group C animals in 2^nd^ half of pregnancy.

This was a blinded randomized controlled study. The study was conducted in accordance with the European Medicines Agency recommendations for clinical trials, with the independent monitoring of the study carried out by Tests and Trials Company, Monzon, Spain. Training on good clinical practices was given to all study participants before commencement of the trial. Female animals were tested for pregnancy by manual palpation and ultrasonography in cattle, and by ultrasonography only in sheep and goats. The animals were then divided into three groups; A, B and C shown in Fig. 1. Group A enrolled males and non-pregnant females allocated to two treatments using a randomised complete block design run at each farm and randomised to treatment (one randomization per farm and species). Group B enrolled cattle, sheep and goats in the 1^st^ half of pregnancy allocated to the two treatments using randomised complete block design in all three farms. Group C enrolled cattle, sheep and goats in the 2^nd^ half of pregnancy allocated to the two treatments using randomised complete block design and run in all three farms on the species available. For each of groups A, B, and C, the placebo and vaccinated animals were housed separately to avoid contact after drug administration. Animals were monitored for 1 year with sampling on days 0, 14, 28, 56, 183 and 365. All study personnel were blinded to the treatment status of the study animals.

### Clinical Assessments of Animals

Rectal body temperature was taken for each animal on day 0, 1, 2 and 3 post-injection. In addition, any animal that developed a clinical condition at any time during the study was subjected to full clinical examination by a veterinarian, specimens collected for analysis, and diagnosis provided. For any animals that died, complete post-mortem investigations were carried out.

### Serology

Sera were collected on day 0, and 14, 28, 56, 183 and 365 days post-injection from each animal and tested for anti-RVF virus IgM and IgG antibodies by enzyme-linked immunoassay (ELISA) using a kits from Biological Diagnostic Supplies Limited (United Kingdom), according the manufacturer’s instructions [33].

For IgG antibody detection, a recombinant antigen-based indirect ELISA was used to detect anti-RVF virus IgG antibodies. Positive sera were detected using recombinant Protein G horseradish peroxidase conjugate and 2,2'-Azinobis [3-ethylbenzothiazoline-6-sulfonic acid]-diammonium salt (ABTS) substrate. Net optical density (OD) values were recorded for each specimen and then used to calculate percent positivity (PP) using a positive control provided by the manufacturer as the denominator. Appropriate negative controls were run with each set of specimens. The PP cutoff was ≥25 for all three species.

For IgM antibodies, an IgM capture ELISA was carried out using rabbit anti-sheep IgM capture antibody and positive specimens detected using mouse anti-RVF virus antibodies and horseradish peroxidase-conjugated anti-mouse IgG. The net OD values of test specimens were expressed as PP of the positive control provided by the manufacturer, with a PP cut-off of ≥7.9 for sheep specimens, ≥ 9.5 for goat specimens, and ≥14.3 for cattle specimens.

#### Virus Neutralization Test (VNT)

Virus neutralization was carried out on serum specimens collected from between 14 and 366 days post-vaccination as described previously [34]. Briefly, duplicates of serial two-fold dilutions of sera inactivated at 56°C for 30 min were tested using microneutralisation procedure as previously described [34]. Titers were expressed as the reciprocal of the serum dilution that inhibited 75% of viral cytopathic effect. A serum sample was considered positive when it had a titer of log10 1.0, equivalent to a serum dilution 1:10. The VNT testing was blinded to the ELISA test results.

#### Statistical analysis

To test whether there were significant statistical differences in seroconversion (ELISA positive) between the vaccinated and placebo treatment groups, logistic regression analysis was carried out on ELISA results of day 14 post-treatment. The outcome variable was sero-status at day 14, and the predictor variable the treatment group to which each animal belonged. Odds ratios of sero-conversion in the vaccine group over the placebo group were calculated using the *epicalc* statistical package [35] on the R platform [36]. Virus neutralization testing (VNT) was carried out on blinded ELISA-positive (N = 130) and ELISA negative (N = 11) samples. The Kappa coefficient was calculated to measure the agreement between ELISA and VNT test results above that which would be expected by chance alone, for all specimens tested using the two tests.

## Results

### Immunogenicity of RVF Clone 13 Vaccine

**Fig 2:**
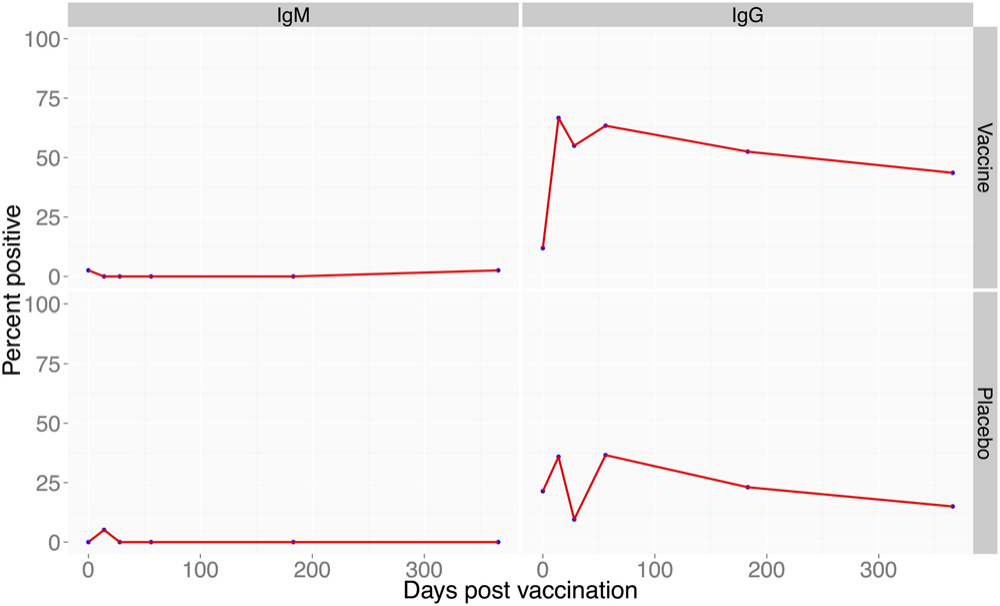
Proportion of cattle positive for anti-RVF antibodies following vaccination with RVF Clone 13 vaccine. Left panel (bottom) shows proportion of cattle with IgG antibodies following vaccination 14 to 366 days post-vaccination. The right panel (bottom) shows proportion of cattle that produced anti-RVF IgM antibodies over the 1 year period. The top panel on the left and right are the placebo-treated animals.

**Fig 3:**
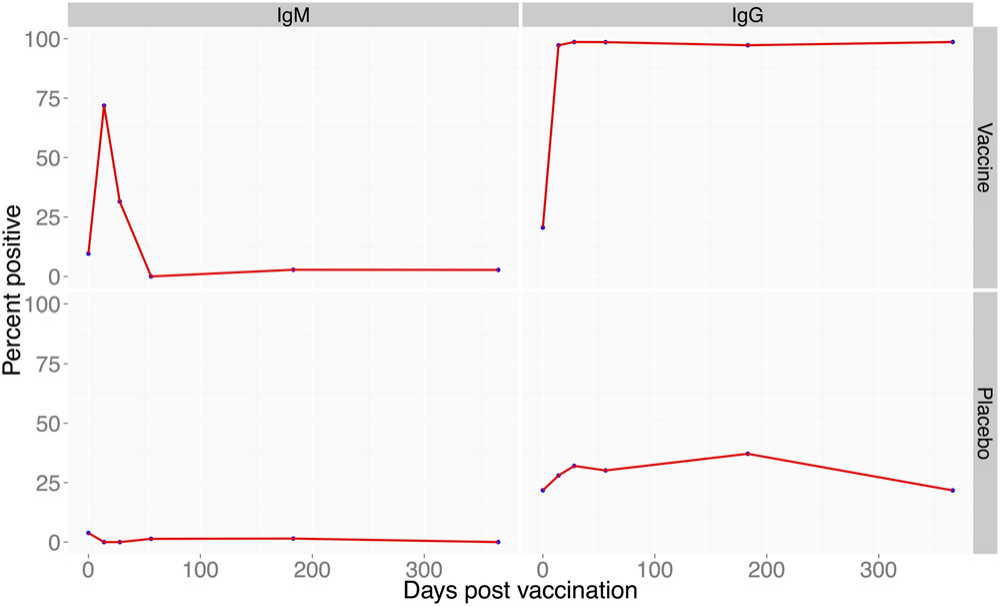
Proportion of goats positive for anti-RVF antibodies following vaccination with RVF Clone 13 vaccine. Left panel (bottom) proportion of the goats with IgG antibodies following vaccination within 14 to 366 days post-vaccination. The right panel (bottom) shows proportion of goats that produced anti-RVF IgM antibodies over the 1 year period. The top panel on the left and right are the placebo-treated animals.

**Fig 4:**
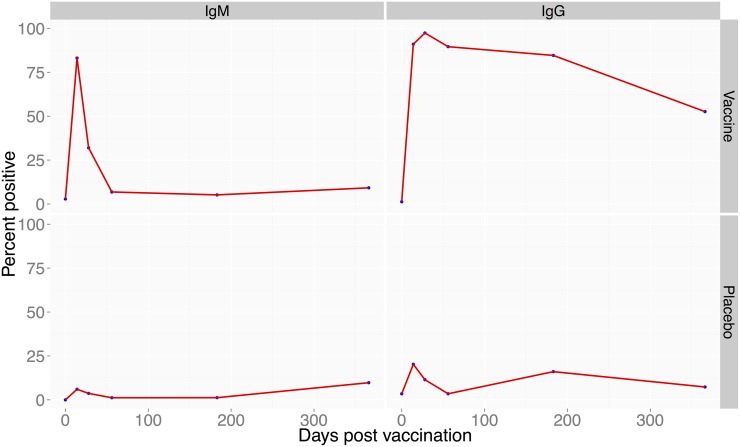
Proportion of sheep positive for anti-RVF antibodies following vaccination with RVF Clone 13 vaccine. Left panel (bottom) shows proportion of sheep showing IgG antibodies following vaccination within 14 to 366 days post-vaccination. The right panel (bottom) shows proportion of sheep that produced anti-RVF IgM antibodies. The top panel on the left and right are the placebo-treated animals.

Among the 3 study farms, a total of 404 animals were enrolled in the study; 151 goats, 85 cattle and 168 sheep. Of these, 170, 87, and 147 animals were enrolled in group A, B and C, respectively (Fig. 1). Vaccinated cattle (N = 42) did not develop any anti-RVF virus IgM antibodies but 67% developed anti-RVF virus IgG antibodies that were maintained in 43.6% of the animals through day 365 post-vaccination (Fig. 2). In vaccinated goats (N = 72), 72% developed IgM antibodies and 97% developed IgG antibodies that were maintained in all animals through day 365 (Fig. 3). In vaccinated sheep (N = 78), 84% developed IgM and 91% IgG antibodies that were maintained in 52.6% of the animals by day 365 (Fig. 4). At day 14, the odds of being seropositive for IgG in the vaccine group were significantly higher (P < 0.05) in vaccinated animals when compared to the placebo group, with an odds ratio of 4 in cattle, 90 in goats, and 40 in sheep (Table 1). The IgM antibody response between placebo and vaccinated groups in cattle and goats was statistically insignificant. However in sheep, IgM response was significantly higher in vaccinated as compared to placebo group of animals (Table 1).

**Table 1:** Odds ratios for sero-conversion by day 14 for vaccinated and placebo groups of cattle, sheep, and goats.

	Odds ratios	Lower 95 CI	Upper 95 CI		*p*-value
Odds of vaccine over placebo					
**IgG**					
Cattle	3.57	1.45		9.16	0.006
Goats	90	25.13		579.21	< 0.001
Sheep	39.97	16.51		110.47	< 0.001
**IgM**					
Cattle	-	-		-	0.997
Goats	-	-		-	0.987
Sheep	84	30.75		282.37	< 0.001

The ratio represents the odds of sero-conversion in the vaccinated group compared to the placebo (reference) group.

### Induction of Neutralizing Antibodies

To determine whether the IgG antibodies were neutralizing, we conducted VNT for vaccinated bovine (N = 40), goats (N = 46), and sheep (N = 46) sera that were ELISA positive. Overall, 89.6% (116/130) of the ELISA positive samples were also positive by VNT whereas 91% (10/11) of the ELISA negative samples were negative by VNT (Kappa = 0.52). Table 2 compares the ELISA titers (expressed as percent positive) against VNT titers (log dilution). As shown previously, cattle had lower ELISA and VNT titers whereas goats had the highest titers (Table 2).

**Table 2:** Comparison of ELISA and VNT titers in RVF Clone 13 vaccinated cattle, goats, and sheep.

Species	N	Mean VNT titer* [SE]	Min, Max	Mean ELISA titer^¶^ [SE]	Min, Max
Cattle	40	24 [3.6]	<10, 80	64 [3.6]	42, 159
Goats	45	267 [34.7]	10, 1280	148 [3.05]	122, 214
Sheep	45	132 [17.1]	<10, 480	104 [2.1]	88, 150

Specimens for VNT were selected from animals vaccinated for between 14 and 366 days.

*expressed as serum dilution,

^¶^expressed as percent positive

### Safety of RVF Clone 13 Vaccine

No fever was reported in any of the study animals. One goat at Kiboko farm was killed by predators and two animals in Ngong farm (a sheep and a cow) died as a result of bloating. There were no RVF-associated mortalities among the study animals. One case of abortion was reported in a vaccinated goat at late pregnancy. However, histopathologic examination and laboratory testing by polymerase chain reaction did not reveal evidence of RVF virus infection. There were no congenital malformations in either the placebo or vaccinated animals.

## Discussion

Although the need for a better RVF livestock vaccine has been recognized for many years, the efforts to generate and manufacture one have been slow and difficult. This is perhaps because of the limited market, which is primarily in Africa, and the fact that RVF cases and epidemics can sometimes be absent for over 10 years even in endemic countries. As a prevention and control measure, livestock vaccination should be used in the context of a national strategy guided by a risk-based analysis [20]. Whether the strategy includes structured regular vaccinations to prevent the disease or immediate action to prevent an imminent outbreak, livestock vaccination needs to be systematic and thorough, targeting all high risk regions in a country and accompanied by other important measures such as monitoring and evaluation to determine coverage and sentinel surveillance (Munyua et al., IN PRESS). The explosive and sequential nature of RVF outbreaks in sub-Saharan Africa has often meant that when an early warning for the disease is issued, even in the presence of adequate resources, there are inadequate vaccine reserves globally to cover livestock at risk in one country, leave alone in multiple countries. To mitigate this risk, the idea of creating a shared vaccine bank consisting of epidemic—prone countries has been discussed but never implemented.

The RVF Clone 13 vaccine had undergone testing under experimental conditions but no studies under field conditions had been conducted (31, 32). Therefore, we undertook this field trial in various commercial livestock farms in Kenya in order to obtain data on safety and immunogenicity under various livestock management conditions that can enable the vaccine to be registered broadly in Africa. Our data shows that RVF Clone 13 vaccine is safe to use in pregnant livestock, with the one abortion in a goat in this study not linked to the vaccine based upon histopathological analysis. The RVF Clone 13 safety finding is in agreement with other studies using this natural mutant that lacks 70% of the NSs gene of the virus, and that has been shown to be avirulent to mice, sheep, goats and cattle in various studies [30–32]. More importantly, the virus has undergone many serial passages in a number of laboratories for over 20 years with no evidence of reversion to virulence *in vitro* or *in vivo*, making it an ideal and safe RVF vaccine that will be acceptable for mass livestock vaccine to farmers during periods of imminent outbreaks. Our data also showed high (>90%) immunogenicity of the vaccine in sheep and goats and moderate (> 65%) immunogenicity in cattle. Even though there was no natural RVF outbreak during the period of the field trials, the Clone 13 vaccinated animals developed high neutralizing antibodies. These results are in agreement with experimental challenge studies with the vaccine in calves and sheep where neutralizing antibodies and prevention from challenge with virulent virus were reported in all vaccinated animal [31,32]. The moderate immunogenicity to Clone 13 vaccine observed in cattle in this study is consistent with a previous study that showed cattle tend to mount a weaker immune response to RVF virus vaccine when compared to sheep and goats [37].

It been suggested that the NSs deletion in RVF Clone 13 vaccine virus gives it capacity for differentiating infected from vaccinated animals. However, we do not have a validated molecular or serological test that can be applied for this purpose. The next steps for RVF Clone 13 are to apply for licensing of the vaccine in Kenya and other African countries. In Kenya, the Department of Veterinary Services is using the data from this study to file for the licensing of the vaccine through Kenya Pharmacy and Poisons Board.

This study had some limitations. First, the use of 1 x 10^5^ pfu dose in cattle, which was informed by controlled experimental studies, was misguided. Therefore, we recommend that for the purpose of registration, the RVF Clone 13 vaccine be administered at 1 x 10^5^ in goats and sheep and a higher does (to be determined) in cattle. Another field study to confirm the safety and immunogenicity of this higher dosage of vaccine is planned. Second, there was no evidence of natural RVF infection in any of the three farms that would have enabled us to assess the ability of RVF Clone 13 vaccine to protect from infection. Third, a few placebo animals were seropositive for anti-RVF IgG antibodies suggesting either mild natural infection during the trial or possible RVF Clone 13 transmission within the herds.
